# Examining the degree to which paranormal belief and conspiracy endorsement influence meaning in life: sequential mediating effects of creativity and self-esteem

**DOI:** 10.3389/fpsyg.2025.1567920

**Published:** 2025-05-08

**Authors:** Neil Dagnall, Andrew Denovan, Kenneth Graham Drinkwater

**Affiliations:** ^1^Manchester Metropolitan University, Manchester, United Kingdom; ^2^Liverpool John Moores University, Liverpool, North West England, United Kingdom

**Keywords:** paranormal belief, conspiracy belief, creativity, self-esteem, schizotypy, latent profile analysis, mediation

## Abstract

Via a shared link with schizotypy, paranormal belief (PB) and conspiracy theory endorsement (CT) influence meaning in life (presence and search). This association is important because meaning in life (particularly presence) is a significant prognosticator of positive wellbeing. Despite this, previous research in this domain remains limited. Major restrictions being the assumption that belief is homogeneous and the failure to consider how factors related to positive wellbeing (i.e., creativity and self-esteem) explain links between belief, schizotypy and psychological health. Accordingly, based on PB, CT, and schizotypy, this study used latent profile analysis (LPA) to identify belief subgroups. Analysis then employed sequential mediation to assess whether creativity and self-esteem mediated the relationship between belief and meaning in life. A sample of 647 completed measures at four time points 2 months apart. At baseline, LPA identified two subgroups: Lower (Profile 1) vs. Higher (Profile 2) belief Ideation. Path analysis revealed that Profile 2 (vs. Profile 1) predicted greater search over time. Moreover, Profile 2 predicted creativity (self-efficacy and personal identity), which in combination with self-esteem, sequentially mediated the belief-meaning in life relationship. Explicitly, creative self-efficacy prognosticated greater self-esteem, which aligned with greater presence and lower search. Creative personal identity demonstrated a negative link with self-esteem but predicted presence and search. Overall, higher scorers in PB, CT, and schizotypy were less driven to search and more likely to possess presence as a function of possessing confidence in their ability to find solutions to problems and self-esteem.

## Introduction

This paper examined the degree to which validation of empirically flawed notions (i.e., scientifically unfounded assertions, SUAs) influenced meaning in life. To establish this, the authors first considered how paranormal belief (PB) and conspiracy theory endorsement (CT) interrelated within participants. The researchers focused on PB and CT because the constructs feature prominently in SUA literature and affiliate differently to meaning in life balance, which is a prognosticator of positive wellbeing (Dagnall et al., 2024). This was an important conceptual advance, since theorists typically study PB and CT in isolation (construct-focused) or assume, based on moderate to strong associations, that belief in one construct predicts endorsement of the other (generic) (Bensley et al., 2022).

Examples of generic classifications are scientifically unsubstantiated beliefs (i.e., the tendency to endorse unverifiable world statements, Mill et al., 1994) and epistemically unwarranted beliefs (i.e., acceptance of viewpoints not supported by credible data and logical reasoning, Lobato et al., 2014). Collectively, such groupings recognize the high-order characteristics of SUAs. Particularly, that high (vs. lower) advocacy is affiliated with reduced critical thinking and increased scientific skepticism. Though both construct-focused and generic approaches have afforded insights into SUAs they possess limitations. Construct-focused studies fail to consider whether outcomes generalize across belief types, whereas the generic approach emphasizes commonality to the exclusion of difference.

Despite generating a robust body of work, these approaches fail to consider how PB and CT interact within individuals. This is important because though correlated, PB and CT differ conceptually and vary in the ways they interact with wellbeing related outcomes. This divergence is commensurate with the constructs distinct features. PB describes authentication of supernatural propositions (e.g., powers, forces, and entities) by individuals who typically engage in rational thinking and reality testing (Irwin, 2009). Conceptually, delimiting CT is difficult since there exists no agreed definition. Accordingly, CT is best explicated as validation of narratives centring on distrust of authority and exploitation of position. Prevailing themes include the ill-intentioned motivations of powerful individuals/groups (e.g., planning, duplicity, intention, and manipulation) (Drinkwater et al., 2023). Validation of both PB and CT is flawed because they derive from a non-scientific and unverified, evidential basis.

The assumption that PB and CT are adaptively similar (i.e., affiliated with wellbeing outcomes) derives from the observation that they relate similarly to cognitive-perceptual factors such as schizotypy and proneness to reality testing errors. The link with schizotypy is especially important since the construct affiliates with a range of psychological health outcomes (Mohr and Claridge, 2015). Noting this, and the fact that schizotypy adds variability to SUAs, recent scholarly work has investigated interactions between, PB, CT, and schizotypy (Denovan et al., 2018; Dagnall et al., 2022b).

Schizotypy is a multidimensional psychopathological construct (Lenzenweger, 2015), which within non-clinical populations theorists regard as a personality dimension ranging from psychological health to schizophrenia (Barrantes-Vidal et al., 2015). In this context, schizotypy designates the extent to which attenuated features of psychotic states occur within healthy individuals (Claridge, 1997). The advantage of this operationalization is that it recognizes that elevated levels of schizotypy exist within general samples without progressing to full spectrum symptoms (Dembinska-Krajewska and Rybakowski, 2014).

Schizotypy comprises positive, negative, and disorganized feature clusters, which map on to factorial models of schizophrenia (Cicero and Kerns, 2010). Positive (productive psychotic-like) symptoms include bizarre perceptions (i.e., hallucinations proneness), thought content disruption (i.e., odd beliefs, magical ideation, and delusion propensity), and suspiciousness/paranoia. Negative (i.e., deficit/restriction) symptoms comprise reduced emotional affect (e.g., flattening, disinterest in external world, and anhedonia). Disorganization symptoms encompass disrupted cognitions and actions. Specifically, thought (structure and expression) and behavior, ranging from mild to formal thought disorder and grossly disorganized actions. These characteristics collectively and at the dimensional level are important because they interact with SUAs. For example, are associated with variations in cognitive performance (Denovan et al., 2018).

Noting that schizotypy can qualify the effects of PB, investigators have combined PB and schizotypy using latent profile analysis (LPA). LPA is a form of mixture modeling that assumes the presence of unobserved (latent) subgroups and probabilistically specifies profiles/class membership using participant responses (i.e., distinct patterns across indicators). The advantage of LPA is that the technique identifies believer subgroups based on multiple variables. This approach has advanced understanding of SUAs by demonstrating that believers are not homogeneous. Instead, belief is heterogeneous, and conviction varies as a function of level of other cognitive-perceptual such as concurrent level of psychopathology (e.g., schizoptypy, Dagnall et al., 2024; manic-depressive experience, Dagnall et al., 2022a) qualify beliefs (Drinkwater et al., 2024).

In terms of wellbeing, this paper focused on meaning in life because the construct is strongly related to psychological and physical health (Czekierda et al., 2017). Specifically, meaning in life is robustly associated with reduced suffering, and better relationships (Steger, 2017). Meaning in life is “the extent to which people comprehend, make sense of, or see significance in their lives, accompanied by the degree to which they perceive themselves to have a purpose, mission, or overarching aim” (Steger, 2009, p. 682). This includes feeling that existence matters and is significant (i.e., has value) (Steger, 2012). Correspondingly, positive psychological theories regard meaning in life as integral to flourishing (i.e., happiness, growth, and optimizing potential) (Diener and Seligman, 2004; Ryff and Singer, 1998) and life satisfaction (Steger, 2017). Concomitantly, individuals with greater meaning in life express greater positivity about themselves and report higher self-esteem, self-acceptance, and positive self-image (Steger et al., 2008).

Researchers regularly assess the construct using the Meaning in Life Questionnaire (Steger et al., 2006), which comprises presence (sense of life as meaningful) and search (drive toward finding meaning). Studies report that these dimensions link divergently with health outcomes. Presence affiliates with positive factors (e.g., life satisfaction), and is inversely related to negative features (e.g., depression) (Steger et al., 2006). Search is associated with reduced wellbeing and negative affect (e.g., sadness and rumination) (Dakin et al., 2021). Though search and presence are independent orthogonal factors, fulfilled search can positively reinforce presence offsetting negative outcomes linked to Search (Newman et al., 2018), whereas unresolved search relates negatively to presence (Russo-Netzer and Icekson, 2023). Moreover, Barnett et al. (2019) found that presence buffered against negative outcomes (psychologist distress, burnout and negative affect) through higher self-esteem. This indicated the vital role that positive affect plays in meaning in life.

Additionally, studies report that creativity enhances meaning in life. Creativity refers to the generation of something novel and useful (Plucker et al., 2004). The positive relationship between the constructs stems from the fact that creativity enables individuals to attain core attributes of meaning of life (i.e., purpose, significance, and coherence) (Kaufman, 2018). Drawing on this interaction, Kaufman (2018) proposed a temporal model where past, present, and future pathways to creativity foster meaning of life. This contends that the past promotes deeper life understanding; the present engages individuals with life and reminds them of enjoyment and connections, and the future provides a sense of legacy and lasting contribution. Regarding self-esteem, González Moreno and Molero Jurado (2023) found that higher levels of creativity were associated with increased self-esteem, suggesting that nurturing creativity increases emotional wellbeing. Additionally, Nemeržitski and Heinla (2020) reported that teachers with higher creative self-efficacy possessed greater self-esteem, which reinforced their creative teaching practices. Added to this, creativity demonstrates a complex relationship with schizotypy. Jacquet et al. (2020) established that positive schizotypy features (e.g., cognitive-perceptual aberrations) aligned with greater creativity than negative and disorganized schizotypy characteristics (e.g., anhedonia, disorganization of thought). This relationship between cognitive-perceptual features and creativity may explain how schizotypy can link with positive wellbeing, such as life meaning.

The present paper used LPA to identify belief subgroups based on PB, CT, and schizotypy scores (baseline). Then employed sequential mediation to assess whether conceptually related wellbeing factors (creativity and self-esteem) mediated the relationship between belief and meaning in life. The researchers selected these factors because they were positively associated and related to meaning in life. The advantage of sequential mediation was that it allowed the authors to examine temporal and causal relationships and capture the directionality of effects over time. This was not possible using a traditional cross-sectional design, which only assesses variables at a single point. Since this study was exploratory, the authors did not state precise hypotheses. Nevertheless, they anticipated that meaning in life scores would vary as a function of subgroup membership and that creativity and self-esteem would mediate the belief subgroup-meaning in life relationship.

## Materials and methods

### Sample

The sample comprised 647 participants (*M*age = 49.9, *SD* = 11.5): 353 males (*M*age = 50.3, *SD* = 10.9), 291 females (*M*age = 49.5, *SD* = 12.2), one trans (age = 49), and two non-binary (*M*age = 37.5, *SD* = 2.1). The researchers recruited participants through Bilendi, an acknowledged supplier of quality data (Kees et al., 2017; Fladerer and Braun, 2020). The researchers asked Bilendi to recruit a representative, gender balanced, UK-based sample (minimum age 18 years). All participants completed measures four times, 2 months apart (i.e., baseline, Time 1, Time 2, and Time 3).

### Measures

The survey employed psychometrically attested self-report measures.

#### Revised paranormal belief scale

The RPBS (Tobacyk, 2004) assessed endorsement of supernatural phenomena (i.e., precognition, psi belief, superstition, traditional religious belief, spiritualism, witchcraft, and extraordinary life forms; see Dagnall et al., 2010). The RBPS contains 26 items presented as statements (e.g., “Black cats can bring bad luck”). Participants record their responses on a 7-point Likert-type scale (1= strongly disagree and 7 = strongly agree). Consistent with Irwin (2009), the researchers recoded responses (0–6). Hence, total scores ranged from 0 to 156, with higher scores indicating greater paranormal belief.

#### Generic conspiracist beliefs scale short

The GCB-5 (Kay and Slovic, 2023) is a short, unidimensional form of the Generic Conspiracist Beliefs Scale (GCBS, Brotherton et al., 2013). Both measures assess the tendency to endorse generic conspiracist ideation. The GCB-5 comprises the highest loading items from each of GCBS factors: government malfeasance, extraterrestrial cover-up, malevolent global conspiracies, personal wellbeing, and control of information. Researchers developed the GCB-5 for use in lengthy test batteries to facilitate participant engagement and completion. Within the GCB measures items appear as statements and participants respond via 5-point Likert-type scale (1 = definitely not true to 5 = definitely true). Total GCB-5 scores range from 5 to 25. Higher totals reflect greater endorsement of general conspiracist notions.

#### Schizotypal personality questionnaire-brief

The SPQ-B (Raine and Benishay, 1995) assesses incidence of normal variability and abnormal degrees of schizotypy using 22 items (e.g., “People sometimes find me aloof and distant”). Participants answer using a dichotomous scale (0 = No and 1 = Yes). The SPQ-B has three subscales, Cognitive-Perceptual Deficits (CP), 8 items; Interpersonal Deficits, 8 items; and Disorganization, 5 items. These correspond with the major symptoms of schizophrenia. CP assesses positive aspects (i.e., odd beliefs and magical thinking, unusual perceptual experiences, paranoid ideation). Interpersonal appraises negative features (i.e., social anxiety, constricted affect, and paranoia). Disorganized evaluates presence of thought disorder and bizarre behavior. In addition to subscale totals, the SPQ-B produces a summative score (0–22). Higher scores signify greater schizotypy.

#### Short scale of creative self

The SSCS (Karwowski, 2012, 2014) appraised trait-like creative self-efficacy (6 items) and personal identity (5 items). Creative self-efficacy refers to an individual's confidence in their ability to produce creative outcomes (e.g., “I know I can efficiently solve even complicated problems?”). Creative personal identity is the perception that creativity is a central part of an individual's self (e.g., “Being a creative person is important to me”). The SSCS presents items as statements and participants record their responses on a 7-point Likert scale (1 = definitely not, 7 = definitely yes). Higher totals specify greater levels of creative self-efficacy and personal identity.

#### Rosenberg self-esteem scale

The RES (Rosenberg, 1965) evaluates global self-esteem (i.e., perceptions of self-worth and acceptance) using 10 statements (e.g., “I take a positive attitude toward myself”). Participants respond using a 4-point Likert type scale (1 = Strongly Disagree to 4 = Strongly Agree). Scores range from 10 to 40 and higher scores designate greater self-esteem.

#### Meaning in life questionnaire

The MLQ (Steger et al., 2006) assessed presence of (5 items, “I understand my life's meaning”) and search (5 items, “I am seeking a purpose or mission for my life”) for purpose in life. Presence is the extent to which individuals believe their being has purpose. Search denotes the degree individuals strive to find or deepen life purpose. Participants indicated endorsement with a 7-point Likert type scale (1 = absolutely untrue to 7 = absolutely true). Subscales range from 5 to 35 and higher scores denote greater presence and search for meaning in life.

Instruments employed within this study have demonstrated robust psychometric integrity (i.e., reliability and validity): RPBS (Drinkwater et al., 2017), GCB-5 (Dagnall et al., 2023; Kay and Slovic, 2023); SPQ-B (Raine and Benishay, 1995); SCCS (Karwowski, 2012, 2014); RES (Rosenberg, 1965); and MLQ (Steger et al., 2006).

### Procedure

The online platform Qualtrics hosted the survey. Participants accessed the site by clicking on the weblink circulated by Bilendi. Prior to entering the survey, participants read the information sheet, which outlined the nature and purpose of the research project. Only participants who provided consent by ticking on the option progressed. The opening survey section consisted of a short demographic section requiring age, preferred gender, and occupation. Participants then progressed to the study scales. To lessen carry-over and order effects the presentation of measures varied across participants. The Qualtrics randomizer controlled this process. Survey instructions directed participants to advance at their own pace and consider items carefully. To counteract social desirability and evaluation apprehension, instructions also informed participants that there were no correct answers and answers should reflect individual inclinations (Krishnaveni and Deepa, 2013). To counter potential common method variance, individual scale instructions emphasized the separateness of sections and construct uniqueness (Spector, 2019). This procedure produces psychological distance between scales and encourages participants to focus on item content. On completion of the measures, the survey delivered the study debrief.

### Ethics statement

The Health and Education Research Ethics Committee at Manchester Metropolitan University provided ethical approval (Project ID, 47784).

## Results

### Analysis plan

The researchers performed statistical analysis using Mplus 8 (Muthén and Muthén, 2015). Data screening and assessment of correlations occurred prior to latent profile analysis (LPA). LPA identified believer subgroups based on Paranormal Belief (PB), Conspiracist Belief (CT), and schizotypy scores. To determine optimal subgroup number the investigators used a likelihood-based significance test. Specifically, the Lo-Mendel-Rubin Adjusted Likelihood Ratio test (LMR-A-LRT; Lo et al., 2001), which compares a k profile with a k-1 profile model. A significant *p*-value specifies a solution requiring additional profiles. Information criteria were Akaike Information Criterion (AIC; Akaike, 1974), Bayesian Information Criterion (BIC; Schwarz, 1978), and Sample-Size Adjusted BIC (ssaBIC; Sclove, 1987). Lower scores on these indicate superior fit.

Next, a path model employed profiles as predictors. This assessed relationships between belief and Meaning in Life (Presence and Search) over time. Within this model, Creativity and Self-Esteem acted as mediators with regards to the belief-Meaning in Life relationship.

Analyses assessed model fit using standard fit indices: Confirmatory Fit Index (CFI), Standardized Root-Mean-Square Residual (SRMR), and Root-Mean-Squared Error of Approximation (RMSEA). Good values are CFI > 0.95, SRMR < 0.05, and RMSEA < 0.05 (Hu and Bentler, 1999). To assess mediation, analyses applied bootstrapping (1000 resamples) to compute 95% bias-corrected confidence intervals (Preacher and Hayes, 2008).

### Data screening

Normality appraisal found acceptable skewness (i.e., between −2.0 and +2.0) (Brown, 2015) and kurtosis (i.e., between −7.0 and +7.0) (Hair et al., 2010). Associations between belief variables (used for LPA) were typical to large (except for Interpersonal with PB, *r* = 0.14) and below 0.8. The latter finding designated absence of multicollinearity (Tabachnick et al., 2013) (Table 1). Consistent with previous literature (e.g., Dagnall et al., 2022b; Denovan et al., 2020), the Cognitive-Perceptual schizotypy factor (vs. Interpersonal and Disorganized) was most strongly associated with PB and CT.

**Table 1:** Descriptive statistics and intercorrelations among paranormal belief, conspiracist belief, and schizotypy.

**Variable**	***Mean***	***SD***	**1**	**2**	**3**	**4**	**5**
1. Paranormal Belief	3.31	1.36		0.63^**^	0.58^**^	0.14^**^	0.31^**^
2. Conspiracist Belief	2.94	0.99			0.52^**^	0.21^**^	0.30^**^
3. Cognitive-Perceptual	0.33	0.29				0.43^**^	0.60^**^
4. Interpersonal	0.52	0.32					0.57^**^
5. Disorganized	0.28	0.30					

^**^ indicates p < 0.001.

### Latent profile analysis

**Figure 1:**
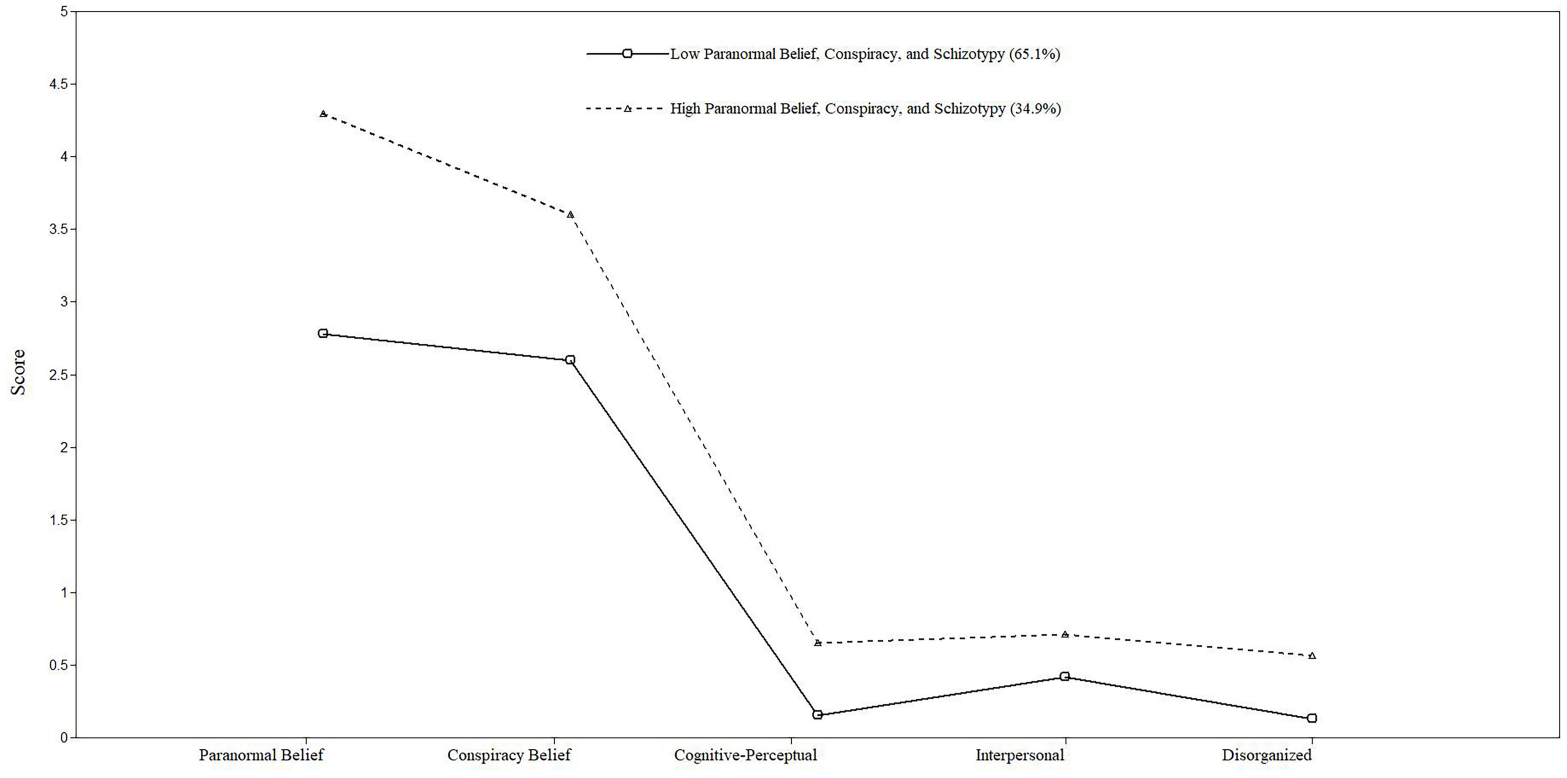
Pattern of scaled mean scores for Paranormal Belief, Conspiracist Belief, and schizotypy.

LPA identified two belief subgroups using a hierarchical approach. This started with one group and then assessed additional groups until a non-significant LMR-A-LRT occurred alongside minimal divergence in AIC, BIC and ssaBIC. The two-profile (vs. one-profile model solution) significantly improved fit (see Supplementary material). A three-profile solution produced a non-significant LMR-A-LRT concomitant with small decreases in AIC, BIC, and ssaBIC. Thus, further analyses adopted the two-profile model (Figure 1).

Profile 1, “Lower Belief Ideation” (65.1% of the sample), displayed lower scores across variables and vs. scale norms, whereas Profile 2, “Higher Belief Ideation” (34.9% of the sample) displayed higher scores across variables and vs. scale norms. Profile 1 (compared with Profile 2) means were PB (4.30 vs. 2.78), CT (3.60 vs. 2.59), Cognitive-Perceptual (0.65 vs.0.15), Interpersonal (0.71 vs. 0.41), and Disorganized (0.56 vs. 0.13).

Since only one high belief profile emerged, path analysis evaluated predictive relationships between Profile 2 (Higher Belief Ideation) and Meaning in Life (Presence, MLPresence; and Search, MLSearch). Creativity (Self-efficacy, CRSE; and Personal Identity, CRPI) and Self-Esteem assessed at Time 2 and Time 3, respectively, functioned as mediators. The initial model was saturated and reported perfect fit. Accordingly, analysis specified a model constraining non-significant predictive paths to zero (i.e., CRSE with both MLPres and MLSearch). This produced a model with good fit, χ^2^ (2, *N* = 647) = 3.94, *p* = 0.139, CFI = 0.99, SRMR = 0.01, RMSEA = 0.03 (95% CI of 0.01–0.09).

**Figure 2:**
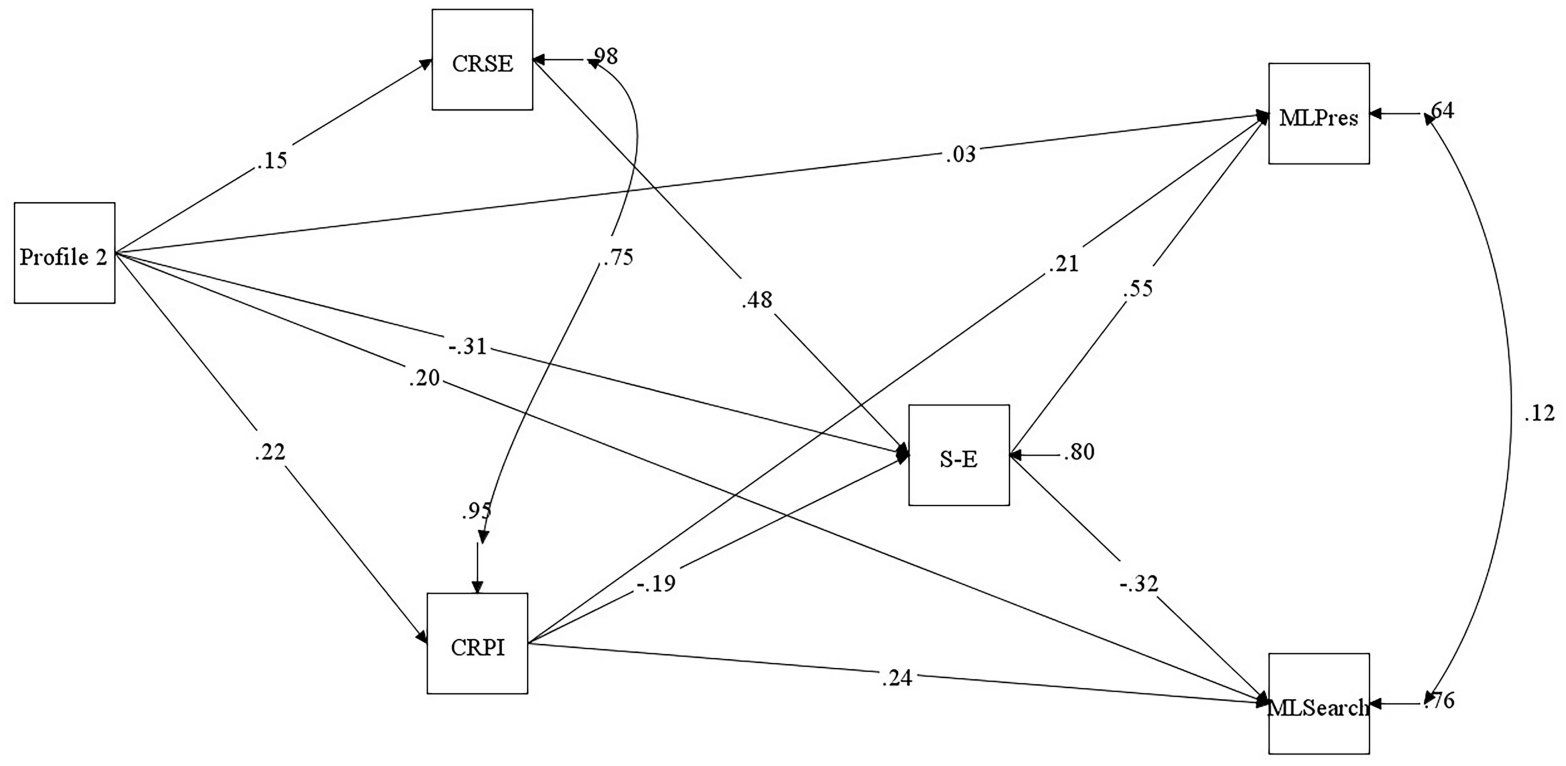
Multiple time point path model portraying relationships between latent profile (reference category = Profile 2), Creativity, Self-esteem, and Meaning in Life outcomes. CRSE, Creativity Self-efficacy; CRPI, Creativity Personal Identity; S-E, Self-esteem; MLPres, Meaning in Life Presence; MLSearch, Meaning in Life Search. Standardized regression weights between variables shown. Error not indicated but specified for all variables. All relationships aside from Profile 2 and MLPres were *p* < 0.05 (using Bootstrapping significance estimates, 1,000 resamples).

Path assessment revealed CRPI and Self-Esteem were positive predictors of MLPres (Figure 2). In addition, CRPI was a positive predictor of MLSearch, whereas Self-Esteem was a negative prognosticator. Vs. Profile 1, Profile 2 exerted a significant indirect effect on MLPresence via CRPI and Self-esteem (Table 2). Profile 2 positively predicted CRPI and negatively predicted Self-esteem; CRPI and Self-esteem positively predicted MLPresence. Profile 2 exerted a significant direct and indirect effect on MLSearch via CRPI and Self-esteem. CRPI positively predicted MLSearch, Self-esteem was a negative predictor. Sequentially, CRPI negatively predicted Self-esteem, and CRSE was a positive predictor, prior to Self-esteem predicting MLPresence (positively) and MLSearch (negatively).

**Table 2:** Specific direct and indirect effects of latent profile on meaning in life outcomes through creativity and self-esteem.

	**MLPresence**	**MLSearch**
**Path**	**β (95%CI)**	**β (95%CI)**
Profile 2	0.03 (−0.03, 0.07)	0.20^**^ (0.15, 0.26)
Profile 2 > CRPersonal Identity	0.05^**^ (0.03, 0.07)	0.05^**^ (0.03, 0.07)
Profile 2 > Self-esteem	−0.17^**^ (−0.20, −0.13)	0.10^**^ (0.06, 0.13)
Profile 2 > CRPersonal Identity > Self-esteem	−0.02^*^ (−0.04, −0.01)	0.01^*^ (0.01, 0.02)
Profile 2 > CRSelf-efficacy > Self-esteem	0.04^*^ (0.02, 0.06)	−0.02^*^ (−0.04, −0.01)

Profile 1 is the reference category; ^*^p < 0.05, ^**^p < 0.001 using Bootstrapping significance estimates (1,000 resamples).

## Discussion

LPA identified two belief subgroups, high (Profile 2) vs. low (Profile 1) belief ideation. Selection of these labels reflected the tendencies of profile members to hold PBs, endorse CTs, and report higher levels of schizotypy, particularly Cognitive-Perceptual deficits. This outcome aligned with the delineation of CP in normal populations as thoughts and behavior that correspond, in an attenuated form, to the positive symptoms of schizophrenia (i.e., odd beliefs and magical thinking, unusual perceptual experiences, paranoid ideation, and ideas of reference). This interpretation was consistent with previous research that reports the presence of these features in endorsers of scientifically unfounded assertions (SUA).

That stated, it is important to acknowledge that feature strength and constituency vary as a function of belief type. Illustratively, CT (vs. PB) is more strongly associated with paranoid ideation (Darwin et al., 2011; Greenburgh and Raihani, 2022) and PB is positively associated with presence of meaning in life, whereas CT is not related (Dagnall et al., 2024). Such differences explain why, although positively correlated, PB and CT shared only approximately 40% variance. In this context, focusing on subgroup commonality, Profile 2 reflected the generalized tendency to validate SUAs.

This paper employed LPA because the method is superior to traditional clustering techniques, which are deterministic (i.e., assign participants to groups without certainty of membership), inflexible (i.e., regard groups as discrete), and perform poorly when data fails to display a spherical or equally sized distribution of clusters. Furthermore, LPA uses indices that specify data fit and prescribe subgroup numbers. For these reasons, the subgroups identified in this study were statistically robust.

Path analysis revealed that Profile 2 (vs. Profile 1) were more likely to possess higher Creativity, lower Self-Esteem and higher Search for Meaning in Life (MLSearch) (i.e., strive to find/deepen life purpose). Moreover, Profile 2 were more likely to possess Presence of Life Meaning (MLPresence) (i.e., think their existence has purpose) due to Creative Personal Identity (CRPI) (i.e., regard creativity as a part of self) and Self-Esteem. These findings concur with previous investigations. Specifically, Bajaj and Lall (2018) who found sense of identity and higher self-esteem contributed to a greater life meaning. Explicitly, the notion that identity and self-esteem interconnect (Stets and Burke, 2014) and contribute to a greater meaning in life (Du et al., 2017). In addition, Profile 2 were more likely to MLSearch (as a function of CRPI) and less likely if they possessed higher Self-Esteem.

A caveat is that Profile 2 (vs. Profile 1) were less likely to possess Self-Esteem, but more likely to index CRPI alongside greater belief in the ability to produce creative solutions (CRSE). Profile 2 possession of higher CRSE was concomitant with higher MLPresence and less inclination to MLSearch due to the positive effect of Self-Esteem. However, CRPI conversely influenced Self-Esteem. These findings demonstrated that Profile 2 (vs. Profile 1) were more creative, but less likely to regard themselves positively. Higher CRPI associated with greater MLPresence and MLSearch. Contrarywise, Profile 2′s affiliation with greater CRSE associated with higher Self-Esteem, which predicted greater MLPresence and lower MLSearch. In terms of previous research, there is limited evidence collectively linking paranormal belief, conspiracy endorsement, and schizotypy to creativity and self-esteem.

However, it is possible that shared cognitive-perceptual features (e.g., magical thinking) between PB, CT, and schizotypy explain associations with higher creativity, which links positively with life meaning via self-esteem. Indeed, research demonstrates that magical thinking is associated with divergent thinking and creativity (Fisher et al., 2004). Moreover, creativity promotes self-esteem and life meaning (Kaufman, 2018; Nemeržitski and Heinla, 2020). Investigators need to undertake additional research to establish the generality of findings and to further explore the complex relationships outlined.

The broad belief classifications identified in the current article (Low 65% vs. High 35%) were consistent with the notion that verification of SUAs occurs frequently within general, non-clinical samples (PB, Dagnall et al., 2016 and CT, Pilch et al., 2023). This coheres with the notion that verification is a typical manifestation of flawed human cognition. Explicitly, the tendency to assume and ratify worldviews based on subjective (internally generated) data (Dagnall et al., 2015; Irwin et al., 2012a,b). In this context, the study of belief is important because beliefs potentially undermine real-world decision making (Bensley, 2023). For example, negatively influence health attitudes/choices and adaptive coping functioning (Denovan et al., 2024a,b). Acknowledging these concerns, ensuing academic work should design interventions that view SUAs holistically, rather than as distinct belief types. The present paper has limitations that merit consideration. Explicitly, while LPA produced statistically appropriate profiles, the identified subgroups lacked a theoretical foundation. This was understandable since the study was novel and exploratory. Also, though profiles were descriptive to the extent that they grouped individuals on the basis of high (vs. low) SUAs and schizotypy, this outcome provides evidence to support the notion that theorists should consider beliefs and allied factors in tandem rather than as isolated constructs (Mill et al., 1994; Lobato et al., 2014).

Additionally, because subgroups identified by LPA arise from cross-variable heterogeneity within particular samples, subsequent studies need to determine whether similar profiles reproduce in independent samples. Establishing subgroup robustness is methodologically and conceptually important. Specifically, it enables theoretical comparison across disparate participant groupings. This is necessary since the investigators drew the present sample from the general population where SUAs and schizotypy scores are typically low and lack variability. Accordingly, subsequent academic work needs to consider a broader range of SUAs and include greater participant diversity (i.e., recruit and compare respondents from general and clinical populations).

To establish equivalence follow-up studies could use crossvalidation methods (Donovan and Chung, 2015). These are statistical techniques such as progressive elaboration that evaluate stability and generalizability by repeating the analysis across different samples and/or data subsets. This iterative process ensures profile robustness across varied conditions and refines profiles by progressively adding detail. Crossvalidation via assessment of profile stability and fit avoids misspecification (Collins et al., 1994). Despite these limitations, the broad subgroupings identified within the current paper provide a sound basis for examining relationships between SUAs and subjective wellbeing.

A further potential limitation in this study arose from the use of self-report measures. Due to deficits in metacognitive awareness, self-report measures are susceptible to cognitive biases. Particularly the Dunning-Kruger effect (Kruger and Dunning, 1999), which occurs when individuals misjudge their competence. This manifests as a mismatch between actual and perceived ability, whereby less proficient individuals overestimate this capacities and more able respondents underestimate their capabilities. In the context of this report, this was problematic with regards to self-efficacy, which Bandura (1997) defined as belief in one's ability to influence personal outcomes, because scores may have reflected perceived (rather than actual) competence. Hence, the illusion of self-efficacy could reinforce presence pf meaning in life in the absence of empirical support.

Self-esteem also influences this process. When based on an inaccurate perceived self-efficacy, strong self-esteem can create a false sense of existential significance. Conversely, low self-esteem may weaken perception of purpose, particularly when personal failures contradict expectations. Additionally, the Dunning-Kruger effect may influence creativity scores. This raises concerns about the accuracy of self-reported creative efficacy and its alignment with objective measures of creative performance. Future research should explore how metacognitive insight influences responses on the Short Scale of Creative Self and whether external, objective validation aligns with self-reported creative efficacy/confidence.

## Data Availability

The raw data supporting the conclusions of this article will be made available by the authors, without undue reservation.
