# Supplementary material for: Examining the degree to which paranormal belief and conspiracy endorsement influence meaning in life: sequential mediating effects of creativity and self-esteem
Source: Front Psychol. 2025 May 8;16:1567920. doi: 10.3389/fpsyg.2025.1567920 (PMC12095287; doi:10.3389/fpsyg.2025.1567920)
Supplement: Supplementary file 1 [file Table_1.docx]

**Supplementary Table 1.** Fit of latent profile models

| Model | AIC | BIC | ssaBIC | LMR-A | LMR-A *p* value |
| --- | --- | --- | --- | --- | --- |
| 1-profile | 5015.27 | 5059.99 | 5028.24 | - | - |
| 2- profile | 4144.73 | 4216.29 | 4165.49 | 860.37 | < .001 |
| 3- profile | 3982.06 | 4080.45 | 4010.60 | 170.29 | .751 |

*Note.* AIC = Akaike Information Criterion; BIC = Bayesian Information Criterion; ssaBIC = sample-size adjusted BIC; LMR-A = Lo-Mendell-Rubin-adjusted likelihood ratio test
